# All-Optical Tuning of Light in WSe_2_-Coated Microfiber

**DOI:** 10.1186/s11671-019-3191-8

**Published:** 2019-11-28

**Authors:** Zhiran Shen, He Zhu, Jiyu Hong, Xun Gui, Heyuan Guan, Jiangli Dong, Hanguang Li, Xiaoli Wang, Wentao Qiu, Enze Zhang, Yunyao Ou, Dongqin Lu, Luqi Luo, Huihui Lu, Wenguo Zhu, Jianhui Yu, Yunhan Luo, Zhe Chen, Gangding Peng

**Affiliations:** 1 Guangdong Provincial Key Laboratory of Optical Fiber Sensing and Communications, Jinan University, Guangzhou, 510632 China; 2 Key Laboratory of Optoelectronic Information and Sensing Technologies of Guangdong Higher Education Institutes, Jinan University, Guangzhou, 510632 China; 3 School of Electrical Engineering & Telecommunications, University of New South Wales, Sydney, Australia

**Keywords:** Tungsten diselenide, Microfiber, All-optical tuning, Thermo-optic effect

## Abstract

The tungsten diselenide (WSe_2_) has attracted considerable interest owing to their versatile applications, such as p-n junctions, transistors, fiber lasers, spintronics, and conversion of solar energy into electricity. We demonstrate all-optical tuning of light in WSe_2_-coated microfiber (MF) using WSe_2_’s broad absorption bandwidth and thermo-optic effect. The transmitted optical power (TOP) can be tuned using external incidence pump lasers (405, 532, and 660 nm). The sensitivity under 405-nm pump light excitation is 0.30 dB/mW. A rise/fall time of ~ 15.3/16.9 ms is achieved under 532-nm pump light excitation. Theoretical simulations are performed to investigate the tuning mechanism of TOP. The advantages of this device are easy fabrication, all-optical control, high sensitivity, and fast response. The proposed all-optical tunable device has potential applications in all-optical circuitry, all-optical modulator, and multi-dimensionally tunable optical devices, etc.

## Introduction

Optoelectronics, photonics, and microelectronics are important and indispensable in modern telecommunication systems. Photonic devices composed of micro- or nanometer-scale optical components are developed to achieve miniaturized structure, fast response, and high sensitivity [1]. Tunable all-optical devices can be applied in optical communication and signal processing. The light-control-light in fiber has been reported, but it remains a challenge to enhance the performance especially the transmitted optical power (TOP) sensitivity and response time. One of the good ways to improve the performance is using the two-dimensional (2D) transition metal dichalcogenides (TMDs), which have been extensively used in the applications of sensors [2], optoelectronic devices [3], transistors [4], saturable absorbers [5], and memory devices [6]. All-optical modulation has been realized with graphene-decorated microfiber (MF) [7], graphene-covered MF [8], and stereo graphene-MF structures [9]. Tuning of MF devices has been achieved when the MF is connected to different materials, such as liquid crystal [10], lithium niobate [11], and polymer [12]. All-optical tunable microfiber knot resonator (MKR) with its top and bottom covered by graphene has been realized [13]. Coating the smooth and lossless surface of the MF with different 2D materials enables light-control-light functionality of MF and MF resonator. All-optical control of light in WS_2_-coated MKR has been reported with a transmitted power variation rate of ~ 0.4 dB/mW under violet pump and a response time of ~ 0.1 s [14]. All-optical light-control-light functionality of MKR coated with SnS_2_ has also been realized; the TOP variation rate with respect to the violet light is ~ 0.22 dB/mW and the response time is as fast as ~ 3.2 ms [15]. The TOP of the MF wrapped with reduced graphene oxide was manipulated by the violet pump light with a variation rate of ~ 0.21 dB/mW [16]. All light-control-light properties of MoSe_2_-coated-MF have also been investigated; the TOP sensitivity is ~ 0.165 dB/mW under violet pump light and the rise time of the transient response is ~ 0.6 s [17]. The TOP sensitivity and response time are important properties of the MF devices. For applications such as all-optical tuning and optical modulation, improvements of the TOP sensitivity and response time are required.

As a typical example of TMDs materials, tungsten diselenide (WSe_2_) has received great research interest, and it is potentially important building blocks for electronic and optoelectronic. WSe_2_ has high Seebeck coefficient, ultralow thermal conductivity, and ambipolarity, making it an attractive candidate for flexible electronics [18, 19]. For example, electrical tuning of p-n junctions has been achieved based on ambipolarity of WSe_2_ [20]. Electrical control of second-harmonic generation in a WSe_2_ monolayer transistor has been reported using strong exciton charging effects in WSe_2_ [21]. WSe_2_ has large absorption coefficient in the visible and near-infrared regions, which has been exploited in conversion of solar energy into electricity [22]. Compared with the sulfide, the selenide is more stable and resistant to oxidation in ambient conditions [23]. In addition, WSe_2_ provides a high intrinsic hole mobility of 500 cm^2^ V^−1^ s^−1^, which is much higher than that of MoS_2_ [24]. Using this property of WSe_2_, high mobility p- and n-type field-effect transistors have been reported with monolayer WSe_2_ [25]. The monolayer WSe_2_ shows a direct bandgap with strong photoluminescence [26]. The nonlinear saturable absorption properties of WSe_2_ have been applied as saturable absorbers in fiber lasers [27]. The WSe_2_ shows great potential for all-optical control of light in WSe_2_-based fiber devices.

The optical MFs are optical fiber tapers with a diameter of several to over 10 μm. The MF is manufactured by simple flame-heated taper drawing the fiber under heat. As a result, the biconical taper is formed proving a platform for interaction between the guided light and the surroundings and connection to other fiberized components [28]. The MF profile can be finely tuned to suit different applications through controlling the pulling speed and time in the fabrication process. The MF has advantages of large evanescent fields, configurability, low optical loss, tight optical confinement, and outstanding mechanical flexibility [29]. The tight optical confinement of MF provides a promising approach to small-footprint optical circuits and low-threshold optical nonlinear effect. Strong and rapid interaction between the guided light and the surroundings can be obtained based on strong evanescent fields of MF. This property of MF has been exploited for optical sensing with different configurations, such as fiber gratings inscribed on MF [30], surface functionalized MF [31], and Mach–Zehnder interferometer [32, 33]. Strong light-matter interaction provided by MF has also been applied to realize all-optical modulator, ultrafast fiber lasers [34, 35], and tuning and light-control-light functionality.

In this paper, we employ the broad absorption bandwidth and thermo-optic effect of WSe_2_ to accomplish all-optical tuning of light in WSe_2_ coated MF. To realize all-optical tuning, the external pump light with wavelengths of 405, 532, and 660 nm are used to irradiate the MF. By employing the interaction between the external pump light and WSe_2_, effective index change is realized and subsequently induces output power variation. The measured TOP sensitivity is 0.30 dB/mW under 405-nm pump light excitation. The external pump laser-induced temperature change and response of the device are investigated. Theoretical simulations are performed to verify the tuning mechanism of TOP.

## Methods

**Fig. 1:**
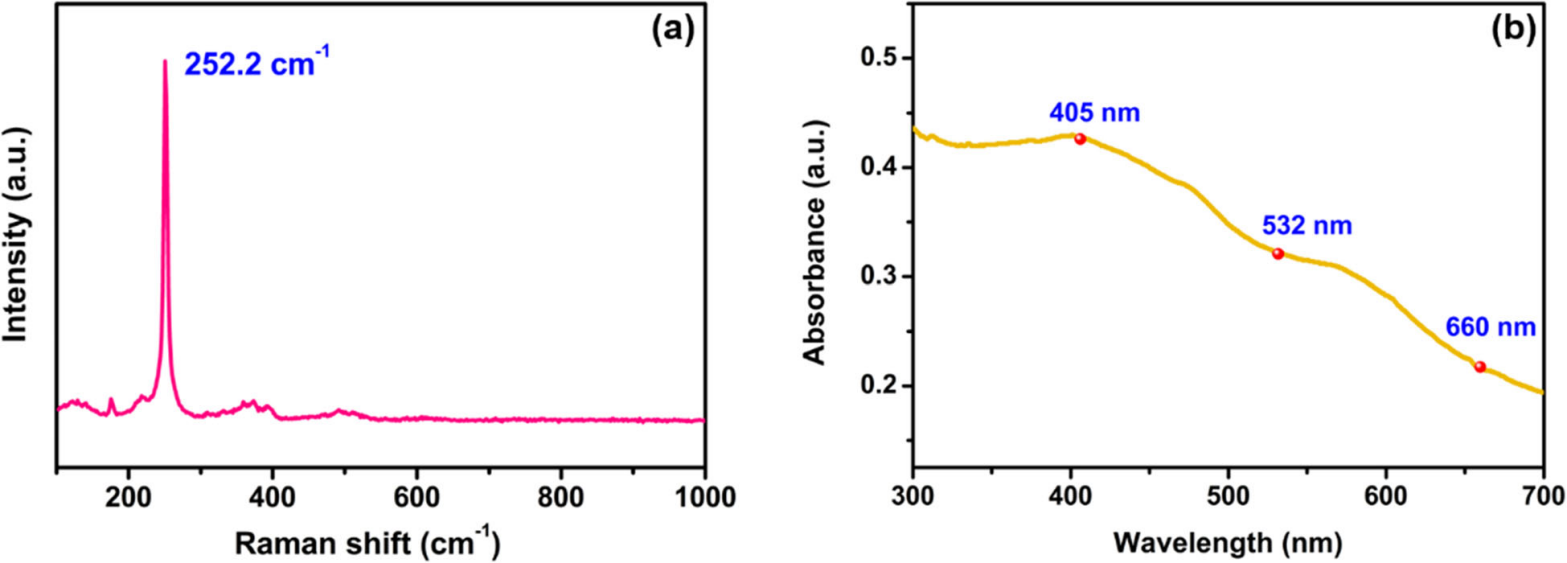
**a** Raman spectrum of WSe_2_. **b** Absorption spectrum of WSe_2_

The concentration of WSe_2_ dispersions was 1 mg/ml, which was obtained through liquid exfoliation method. In order to obtain WSe_2_ nanosheets with uniform distribution, ultrasonic treatment of the WSe_2_ dispersions for ~ 30 min was performed. In order to characterize the WSe_2_ nanosheets, Raman and UV-VIS absorption spectrum were measured. The Raman spectrum of WSe_2_ nanosheets excited by a 488-nm laser is shown in Fig. 1a. The WSe_2_ nanosheets display only one strong vibrational mode around 252.2 cm^–1^, which is a result of degeneracy of the E_2g_ and A_1g_ modes. An additional Raman peak will appear at 5–11 cm^−1^ when the WSe_2_ flakes are thinner than four layers [36]. The absorption spectrum of WSe_2_ nanosheets measured by an UV–VIS spectrophotometer (UV–2600, SHIMADZU) is shown in Fig. 1b. In the wavelength range from 300 to 700 nm, the WSe_2_ nanosheets have absorption. From 400 to 700 nm, the absorption decreases with wavelength. The absorption at three wavelengths 405, 532, and 660 nm is compared, as shown in Fig. 1b.

**Fig. 2:**
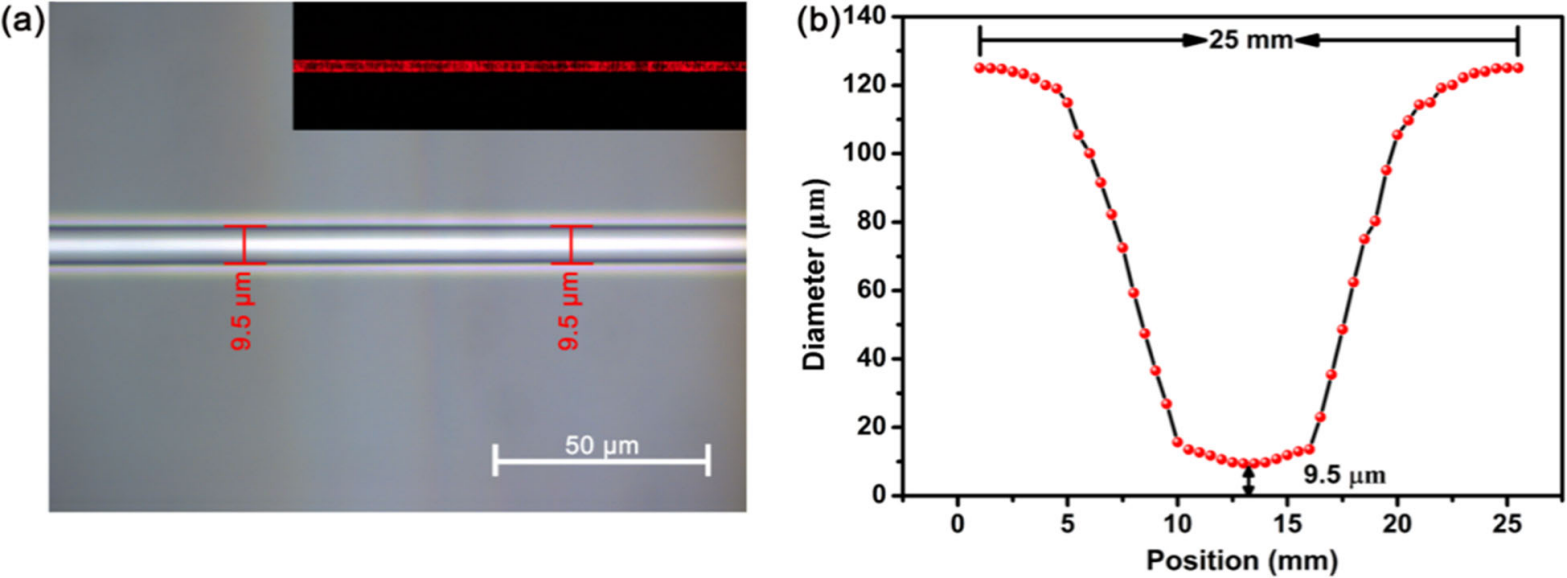
**a** Microscopic image of the fabricated MF. **b** Morphological characteristic of MF

The MF was manufactured using the “flame-brushing” technique. The MF was obtained by drawing a piece of a standard single mode fiber from Corning Inc. at a speed of ~ 0.2 mm/s, heated by a flame. In order to realize all-optical control of light in WSe_2_-coated MF, appropriate waist of the MF is required. A smaller MF waist enables stronger interaction between light and WSe_2_, but the TOP might be too weak to be detected since the loss is large. Figure 2 a shows the fabricated MF with a diameter of ∼ 9.5 μm in the uniform waist region. The inset of Fig. 2a is the microscopic image of the MF with a 650-nm laser launched at the input. The diameter of the MF was measured using an optical microscope (Zeiss Axio Scope A1 microscope). As shown in Fig. 2b, the waist region of the MF has a length of ∼ 6 mm and a diameter of ∼ 9.5 μm. The total length of the MF is ∼ 25 mm.

**Fig. 3:**
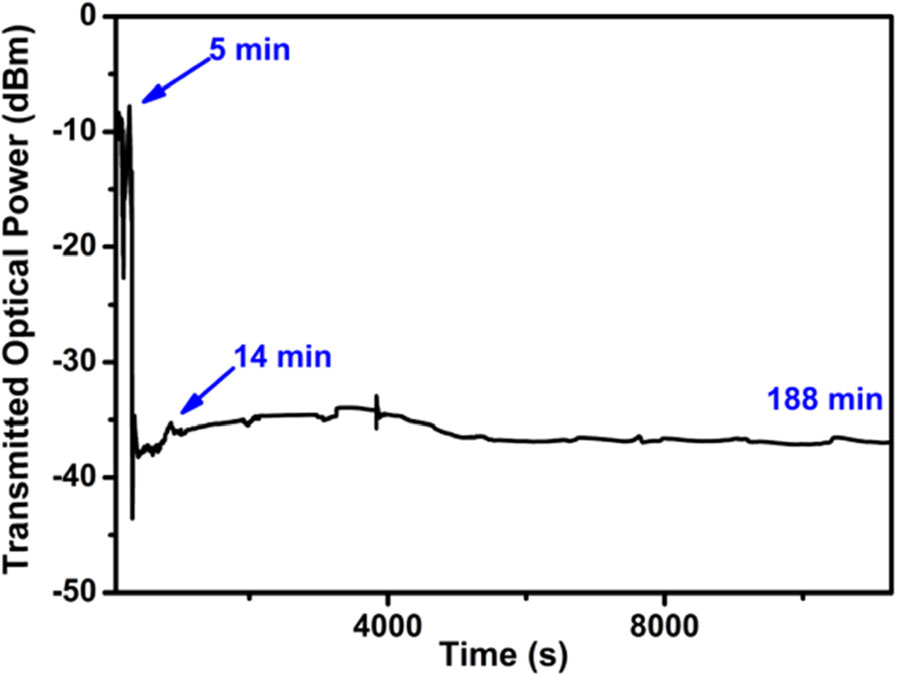
Variation of TOP in MF during the deposition of WSe_2_

Next step was deposition of the WSe_2_ nanosheets onto the MF. Before the deposition, the MF was fixed to a glass basin (20 mm × 5 mm × 1 mm) which was made from glass and UV adhesive (Loctite 352, Henkel Loctite Asia Pacific). After that, the WSe_2_ dispersion was dropped onto the MF using a pipette. The TOP of MF during the deposition process was monitored using a 1550-nm distributed feedback (DFB) laser. As shown in Fig. 3, before deposition, the TOP is about − 10 dBm. After 5 min of deposition, the TOP decreases sharply to − 43 dBm. Then the TOP increases to − 35 dBm after 14 min. The TOP becomes stable at − 37 dBm, indicating the deposition is completed.

**Fig. 4:**
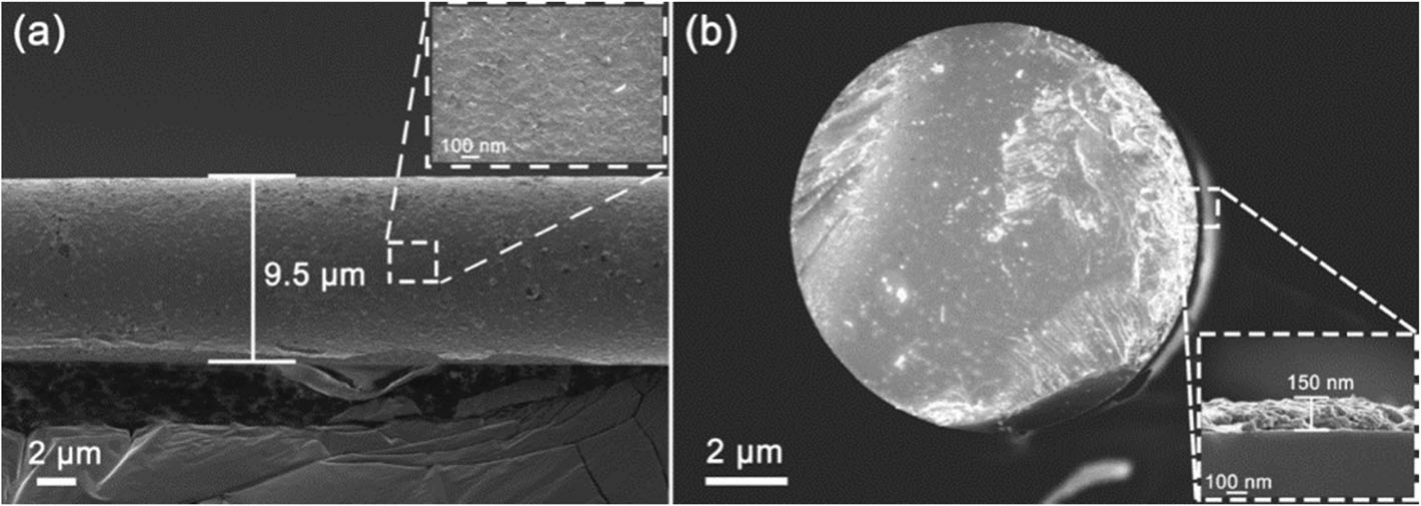
**a** SEM image of the MF coated with WSe_2_. **b** Cross section view of the MF coated with WSe_2_ nanosheets

The scanning electron microscopy (SEM) image of the MF coated with WSe_2_ nanosheets is shown in Fig. 4. Figure 4 a shows the WSe_2_ nanosheets precipitate on the MF with a diameter of ~ 9.5 μm, and its enlarged image is shown in the inset of Fig. 4a. The cross section view of the MF coated with WSe_2_ nanosheets is shown in Fig. 4b. The inset of Fig. 4b shows that the thickness of deposited WSe_2_ nanosheets is ~ 150 nm.

**Fig. 5:**
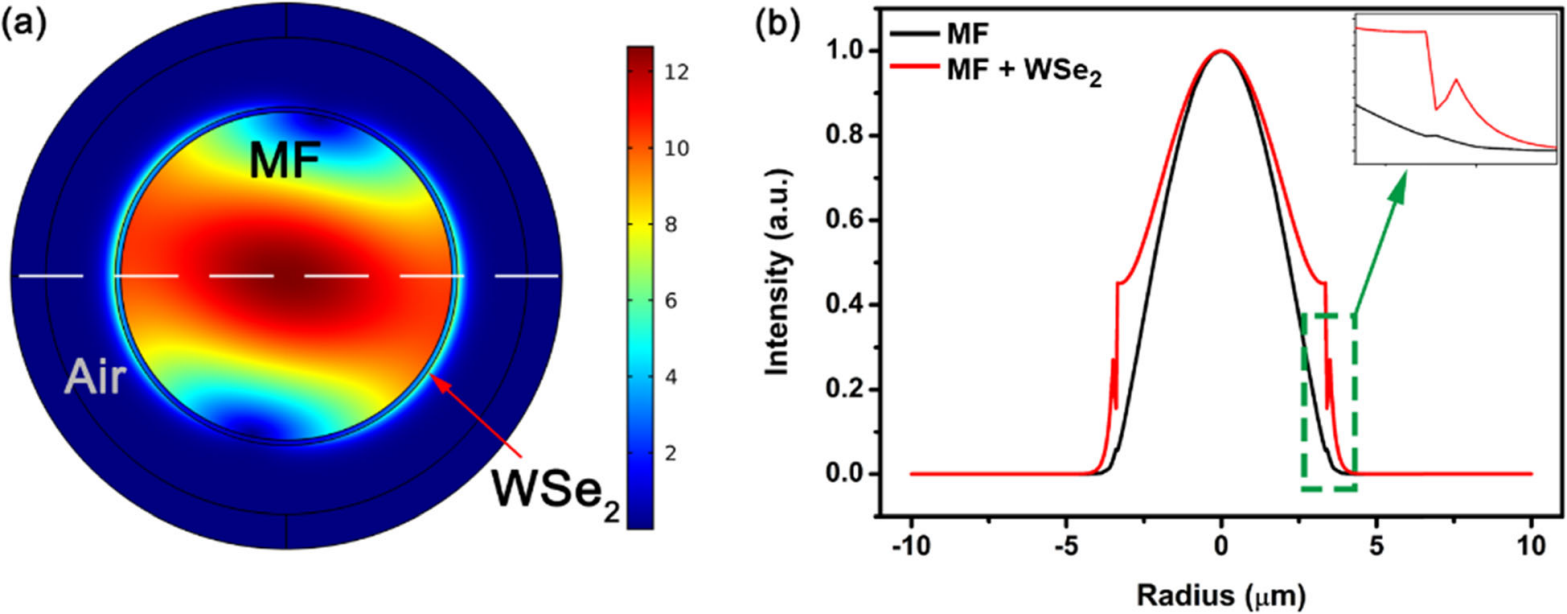
**a** 2D field distribution of the simulated guiding mode in WSe_2_-coated MF. **b** Radial field distribution of the bare MF and WSe_2_-coated MF, and the inset shows a zoomed-in image of the field at the MF surface

To investigate light absorption of the WSe_2_ film, light guiding in the WSe_2_-coated MF was simulated by finite element method in COMSOL. In the model, a 150-nm WSe_2_ layer is wrapped around the ~ 9.5 μm MF. The refractive indices of the MF and WSe_2_ nanosheet are 1.46 and 2.64 + 0.2i [37], respectively. The calculation window is 20 μm × 20 μm and the meshing size is 50 nm. The wavelength was fixed at 1550 nm. The mode field distributions of the MF and the WSe_2_-coated MF were calculated. Figure 5 a shows the 2D mode distribution at 1550 nm. The effective index of the mode in the MF with the WSe_2_ layer corresponding to Fig. 5a is 1.4567–2.04 × 10^−3^ i, indicating WSe_2_’s absorption. The radial field distribution of the bare MF and WSe_2_-coated MF along the white dashed line of Fig. 5a is plotted in Fig. 5b. The radial field distribution has the same peak intensity at ~ 0 μm. In the zoomed–in image of Fig. 5b, the field distribution of WSe_2_-coated MF shows an abrupt variation as a result of index discontinuity.

**Fig. 6:**
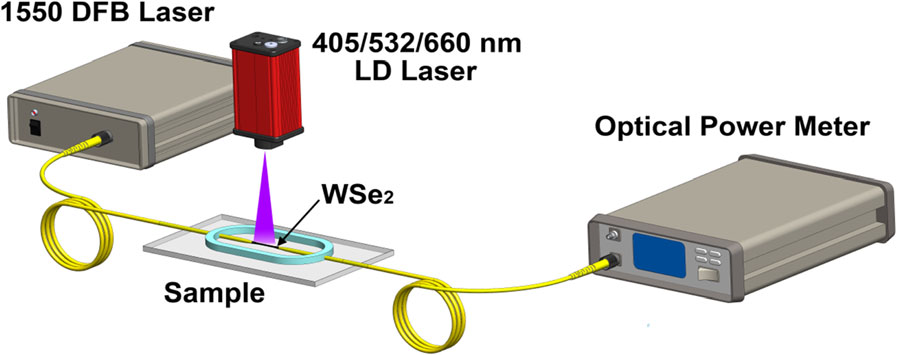
Experimental setup for measuring TOP of the device under external laser light illumination

The all-optical control of light in WSe_2_-coated MF is characterized using the experimental setup as shown in Fig. 6. The 1550-nm DFB laser (SOF–155–D DFB LASER SOURCE, ACCELINK) is connected to the input of the device, and the output is monitored by the optical power meter. The 405-, 532-, and 660-nm lasers are used for external pump. The MF coated with WSe_2_ is irradiated by the lasers that are placed ~ 10 cm above the sample. Firstly, the TOP of MF without WSe_2_ is measured using this experimental setup.

## Results and Discussion

**Fig. 7:**
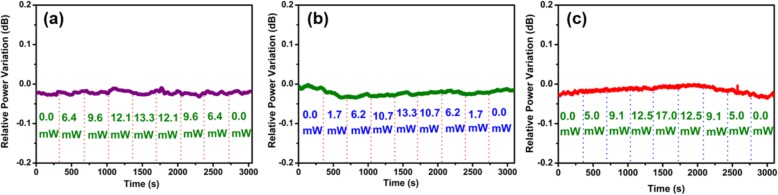
TOP changes with different pump powers under **a** 405-nm laser, **b** 532-nm laser, and **c** 660-nm laser illumination

Figure 7a–c shows the relative power variation for various pump powers of the 405-, 532-, and 660-nm lasers, respectively. As shown in Fig. 7a, the changes of TOP of the bare MF are smaller than 0.03 dB under 405-nm laser irradiation. Similar results are obtained for the 532- and 660-nm lasers. The TOP variations are smaller than 0.02 and 0.03 dB for the 532- and 660-nm lasers, respectively.

**Fig. 8:**
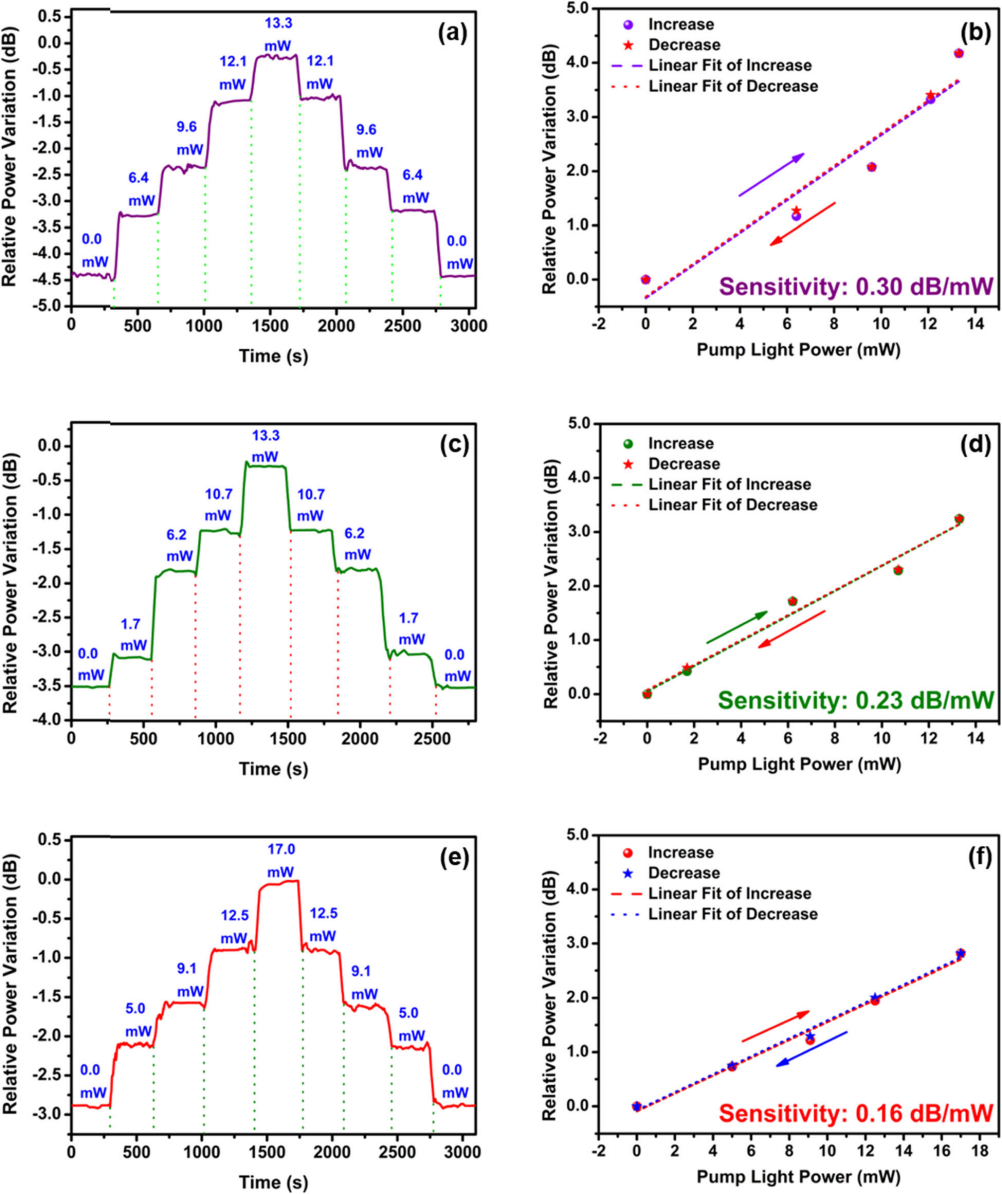
**a** TOP variation under different 405-nm laser power. **b** TOP variation versus 405-nm pump light power. **c** TOP variation under different 532-nm laser power. **d** TOP variation versus 532-nm pump light power. **e** TOP variation under different 660-nm laser power. **f** TOP variation versus 660-nm pump light power

Then the TOP of the MF coated with WSe_2_ nanosheets is measured under different pump powers. The experiments are performed with the 405-nm (violet) laser power (LSR405NL, Lasever Inc.) ranging from 0 to 13.3 mW. Figure 8 a plots the relative power variation of the MF coated with WSe_2_ nanosheets under 405-nm laser illumination. The TOP increases with the pump power. When the pump power of 405-nm laser increases from 0 to 13.3 mW, the TOP variation is 4.2 dB. The TOP variation is also 4.2 dB when the 405-m laser power decreases from 13.3 to 0 mW. In order to analyze the relationship between the TOP and 405-nm laser power, the mean values of the TOP for different steps of pump power in Fig. 8a are extracted. The change of TOP with pump light power is shown in Fig. 8b. The sensitivity of TOP variation to pump power is determined by the slope of linear fitting curve. A sensitivity of 0.30 dB/mW is obtained for both increasing violet power and decreasing violet power, verifying the all-optical control of light has good repeatability and stability. The all-optical control of light of the MF coated with Wse_2_ nanosheets is analyzed with the 532- and 660-nm lasers. Figure 8 c presents the TOP variation when the 532-nm (green) laser power increases from 0 to 13.3 mW. The TOP changes with the green laser power. The relative power variations are 3.2 dB for both increasing pump power (from 0 to 13.3 mW) and decreasing pump power (from 13.3 to 0 mW). The TOP variation for different pump light power is plotted in Fig. 8d. The sensitivities are 0.23 dB/mW for both the increase and decrease processes. Similar results are obtained for the 660-nm (red) laser pump. As shown in Fig. 8e, the TOP increases by 2.9 dB when the red laser power increases from 0 to 17.0 mW, and the power change is the same for the decrease process. The sensitivities under the red laser illumination are obtained from Fig. 8f, which are 0.16 dB/mW for both the increasing pump power (from 0 to 17.0 mW) and decreasing pump power (from 17.0 to 0 mW). In Fig. 8b, d, and f for all-optical tuning, the linearity is different. During the increasing power process, the *R*^2^ values are 0.907, 0.976, and 0.984 for the violet, green, and red lasers, respectively. The *R*^2^ values of 0.915, 0.977, and 0.991 are obtained in the decreasing power process for the violet, green, and red lasers, respectively. Here, the violet laser provides better sensitivity but the linearity of the red laser is better. However, for all-optical control of light in MoSe_2-_coated MF, the 980-nm light has better linearity and sensitivity than the 405-nm light [17]. Therefore, there is no consistent relationship between linearity and sensitivity for different devices under different pump lasers. We believe the linearity and sensitivity are related to the 2D material, the deposition method, the fiber structure, and the stability of the pump light.

**Fig. 9:**
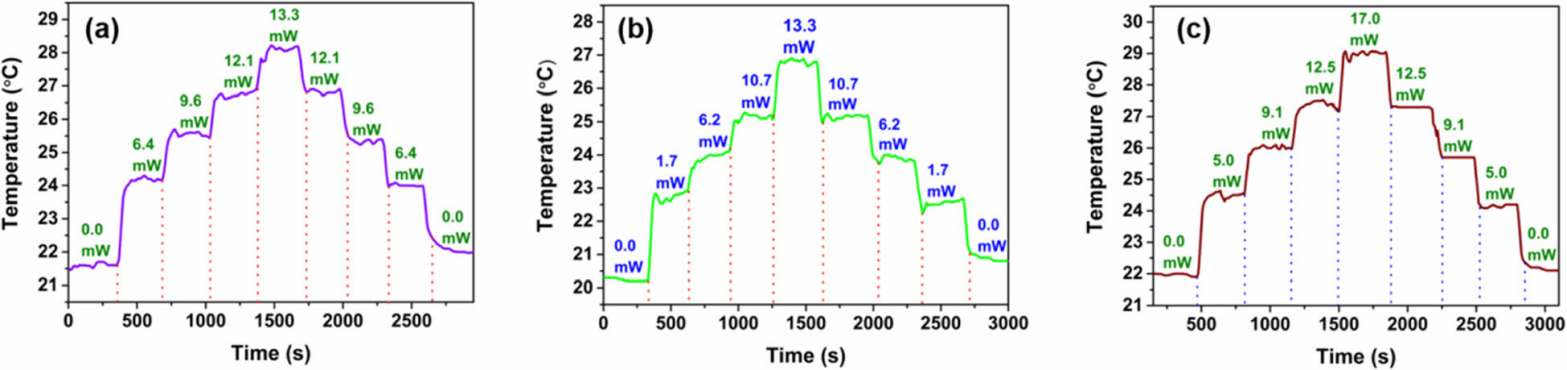
Temperature of the MF coated with WSe_2_ nanosheets for different **a** violet pump power, **b** green pump power, and **c** red pump power

**Fig. 10:**
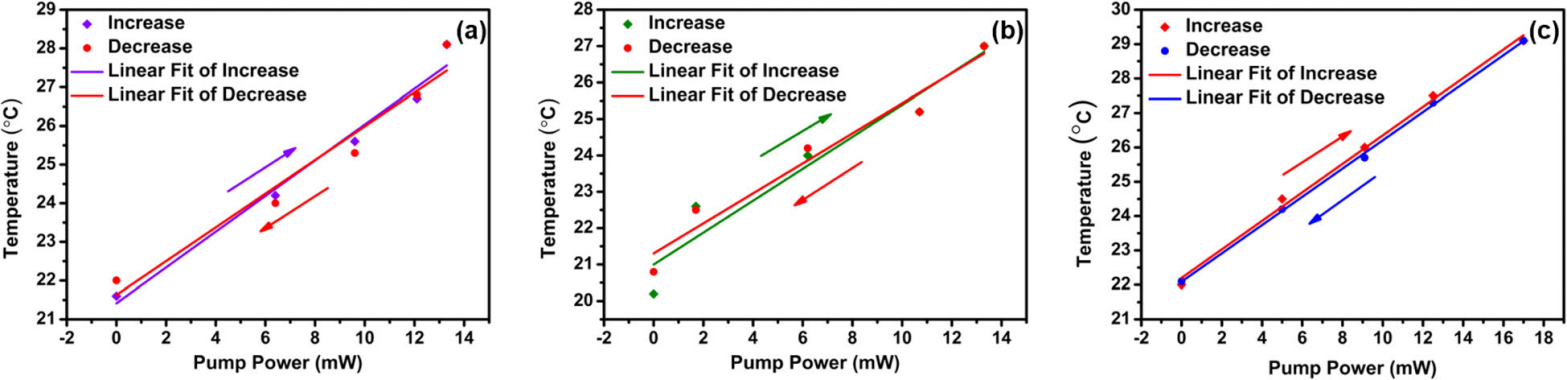
Temperature as a function of pump power for **a** violet laser, **b** green laser, and **c** red laser

**Fig. 11:**
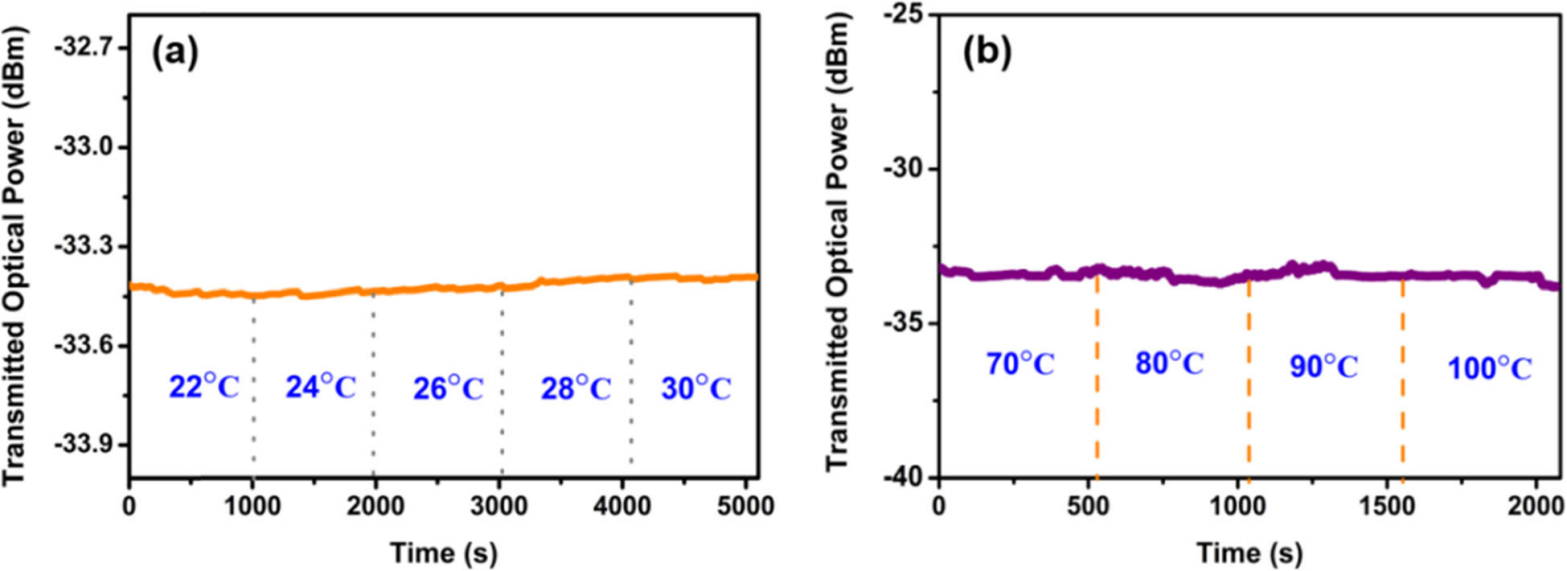
TOP of the MF coated with WSe_2_ nanosheets under **a** different environment temperature and **b** high temperature

It should be noted that the temperature of the MF coated with WSe_2_ changes under laser illumination. The temperature is recorded by a thermocouple when the pump power changes. Figure 9 a shows the change of temperature for various violet pump powers. The temperature increases with the pump power. The temperature increases from 21.6 to 28.1 °C when the violet pump power increases from 0 to 13.3 mW. When the violet pump power decreases from 13.3 to 0 mW, the temperature decreases from 28.1 to 22.0 °C. The temperature variations are also monitored for the green and red pump lasers. As shown in Fig. 9b, increasing and decreasing the green laser powers in the range from 0 to 13.3 mW can induce 6.7 °C and 6.1 °C temperature variations, respectively. Figure 9 c shows the temperature variation under red laser pump, which has the same varying trend. The temperature changes by 7.1 °C and 7.0 °C when the red pump power varies between 0 and 17.0 mW. The temperature as a function of pump power is plotted in Fig. 10. As shown in Fig. 10a, the linear fit of the temperature variation gives sensitivities of 0.46 °C/mW and 0.44 °C/mW for increasing and decreasing violet pump power, respectively. Figure 10 b shows the temperature sensitivities which are 0.44 °C/mW and 0.41 °C/mW for increasing and decreasing green pump power, respectively. For the red pump power increase and decrease process, the temperature sensitivities are measured to be 0.41 °C/mW. The results indicate the WSe_2_ can be regarded as efficient and compact heaters for all-optical control and thermo-optic tuning [38]. In order to investigate the influence of environment temperature on the device performance, the MF coated with WSe_2_ nanosheets is placed onto a ceramic hotplate (CHP–250DF, AS ONE) for TOP measurement. As shown in Fig. 11a, the TOP variations are smaller than 0.03 dB when the chamber temperature is changed from 22 to 30 °C. The results which verify this device is insensitive to the environment temperature. As shown in Fig. 11a, the TOP variations are smaller than 0.03 dB when the chamber temperature is changed from 22 to 30 °C. The results which verify this device is insensitive to the environment temperature. This device is relatively stable when it is used in high temperature for all-optical tuning. As shown in Fig. 11b, when the temperature is increased from 70 to 100 °C slowly, the TOP variations are smaller than 0.55 dB.

**Fig. 12:**
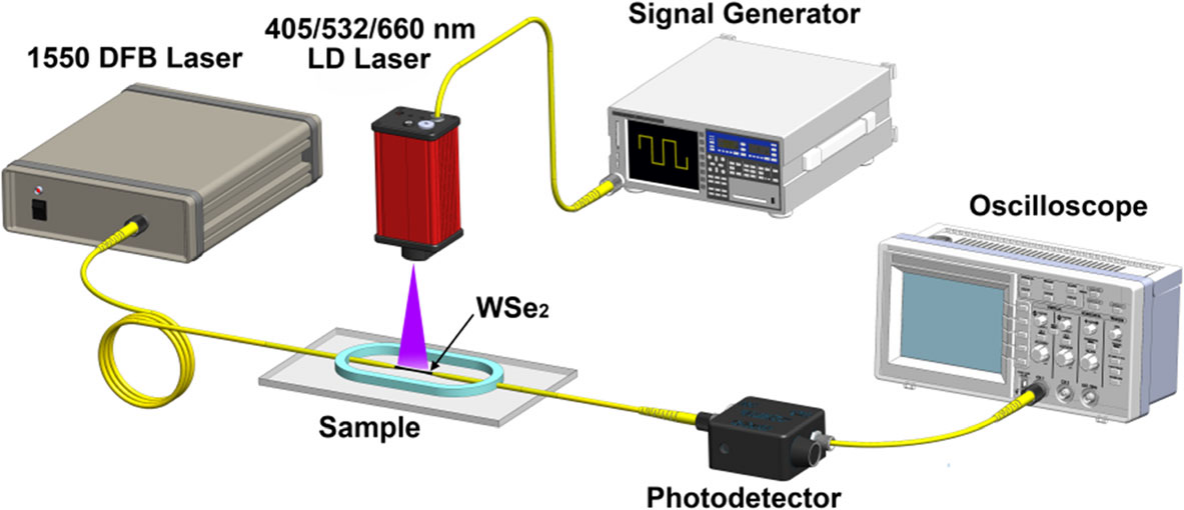
Experimental setup of the transient response measurement

**Fig. 13:**
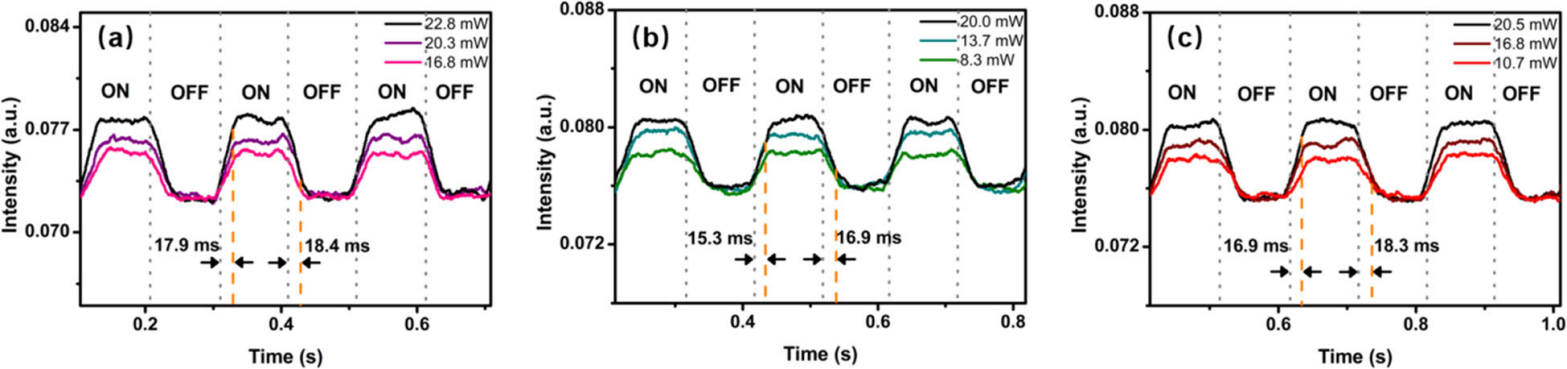
Response time of the MF coated with WSe_2_ nanosheets with a pump light wavelength of **a** violet laser illumination, **b** green laser illumination, and **c** red laser illumination

The transient response of the MF coated with WSe_2_ nanosheets is measured using the experimental setup shown in Fig. 12. The 1550-nm laser is connected to the input of the MF. The outputs of the violet, green, and red lasers are modulated by a signal generator (AFG 3102, Tektronix). The output of signal generator is a square wave. A photodetector (Model 1811, New Focus) and an oscilloscope (DS1052E, RIGOL) are used to monitor the output of the MF. Figure 13 a–c shows the response monitored by the oscilloscope under violet, green, and red laser illumination, respectively. As shown in Fig. 13a, the violet pump powers are 16.8, 20.3, and 22.8 mW for response time measurement. The rise time and fall time are measured to be 17.9 and 18.4 ms for the violet laser, respectively. For green laser illumination, the pump powers are 8.3, 13.7, and 20.0 mW, as shown in Fig. 13b. The rise time and fall time are measured to be 15.3 and 16.9 ms for the green laser, respectively. As shown in Fig. 13c, under red laser illumination with pump powers of 10.7, 16.8, and 20.5 mW, the rise time and fall time are 16.9 and 18.3 ms, respectively.

**Fig. 14:**
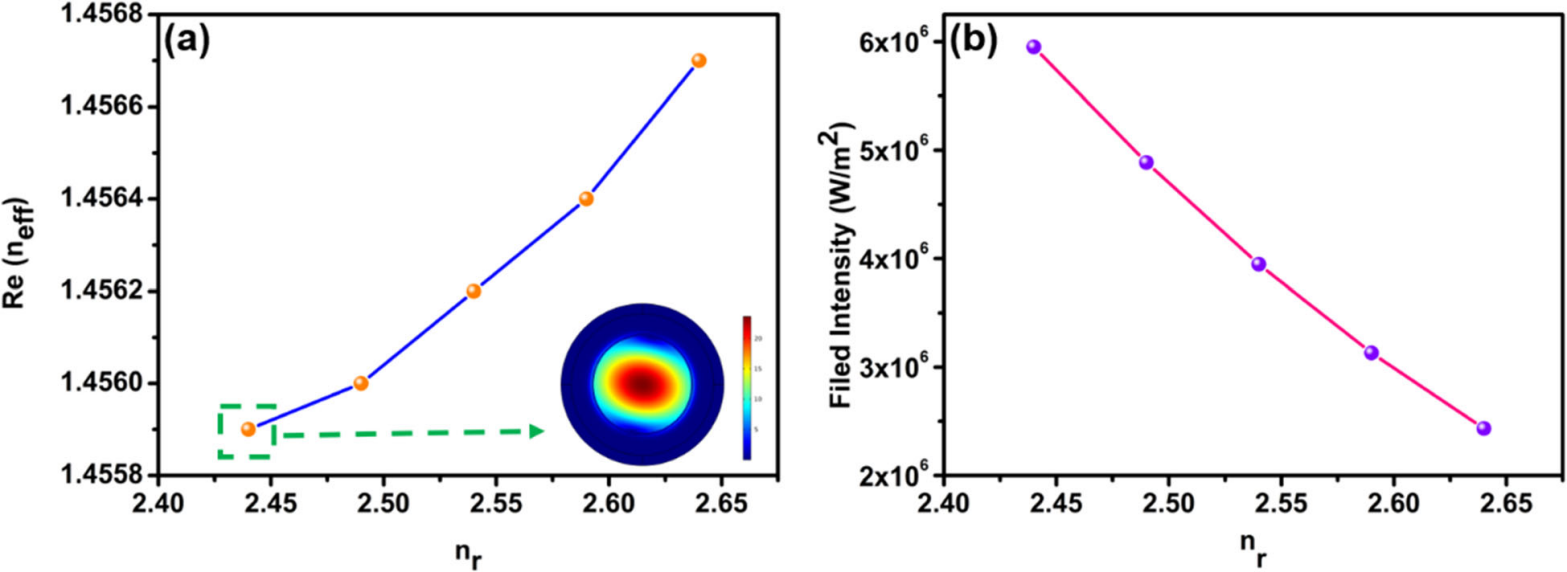
**a** The mode real part of *n*_eff_ as a function of *n*_*r*_ for 150-nm WSe_2_ nanosheet. And the inset is the electric field distribution of the mode with *n*_eff_ of 1.4559. **b** Dependence of output electric energy on *n*_*r*_ for 150-nm WSe_2_ nanosheet

**Fig. 15:**
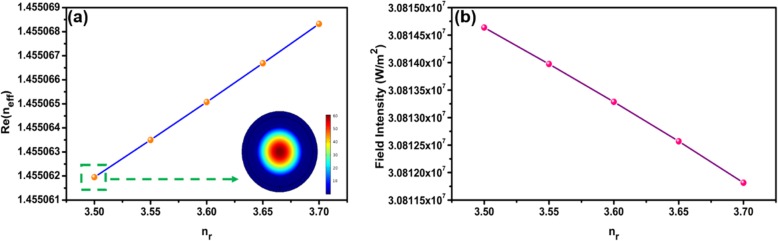
**a** The mode real part of *n*_eff_ versus *n*_*r*_ for the four-layer WSe_2_ nanosheet. And the inset is the electric field distribution of the mode with *n*_eff_ of 1.4550619. **b** Dependence of output electric energy on *n*_*r*_ for the four-layer WSe_2_ nanosheet

The tuning sensitivity of TOP is different for the violet, green, and red pump lasers. This is because the absorption is much stronger at shorter wavelengths, as shown in Fig. 1b. The all-optical control of TOP is due to the combination of thermo-optic effect and photon-generated carriers in MF with WSe_2_. The interaction between the external pump light and WSe_2_ induces effective index change of WSe_2_. The WSe_2_ nanosheets absorb the pump laser light. The temperature of MF with WSe_2_ increases with the pump power, as shown in Figs. 9 and 10. The real part of refractive index (*n*_*r*_) of WSe_2_ decreases when the temperature of the MF with WSe_2_ increases [39]. The *n*_*r*_ also decreases due to the increase of carrier concentrations which is related to conductivity of WSe_2_ nanosheets [40]. As a result, the effective refractive index (*n*_eff_) of guided modes in MF coated with WSe_2_ is varied by external laser illumination. The photon-generated carriers also lead to index variation of WSe_2_ and change of the *n*_eff_ [38]. Therefore, the TOP can be changed with external pump lasers. Using the finite element method, simulations are performed to investigate the mechanisms of TOP tuning. As shown in Fig. 14a, the real part of *n*_eff_ increases with *n*_*r*_. The real part of *n*_eff_ increases from 1.4559 to 1.4567 with *n*_*r*_ varying from 2.44 to 2.64 [41, 42]. The electric field distribution of the mode with *n*_eff_ of 1.4559 is shown in the inset of Fig. 14a. Variation of *n*_*r*_ provides different mode electric field distributions. Integrating the electric field distribution of the whole cross section, the output electric energy is calculated. As shown in Fig. 14b, the output electric energy decreases with *n*_*r*_ from 2.44 to 2.64 with a rate of 1.76 × 10^7^ W/m.^2^ Therefore, the output power increases with the external pump power. The simulation results agree well with the experimental results. In order to investigate the impact of the WSe_2_ layer number on the device performance, simulations were performed by finite element method in COMSOL. The thickness of the four-layer WSe_2_ nanosheet is 2.8 nm, and the corresponding refractive index of WSe_2_ is 3.7 + 0.2i [43]. The linear fit of real part of *n*_eff_ versus *n*_*r*_ is shown in Fig. 15a. The real part of *n*_eff_ increases with *n*_*r*_ when it is varied from 3.50 to 3.70. The electric field distribution of the mode for *n*_eff_ of 1.4550619 is shown in the inset of Fig. 15a which is circularly symmetric. In comparison, the electric field distribution of the mode in Fig. 14a is asymmetric since the light is absorbed by the 150-nm WSe_2_ nanosheet. The output electric energy decreases when *n*_*r*_ increases from 3.50 to 3.70 with a rate of 1.41 × 10^4^ W/m^2^, as shown in Fig. 15b. The output electric energy variation rate of the 150 nm WSe_2_ nanosheet is much larger than that of the 2.8-nm WSe_2_ nanosheet, indicating the thick WSe_2_ nanosheet provides better performance for all-optical tuning.

**Fig. 16:**
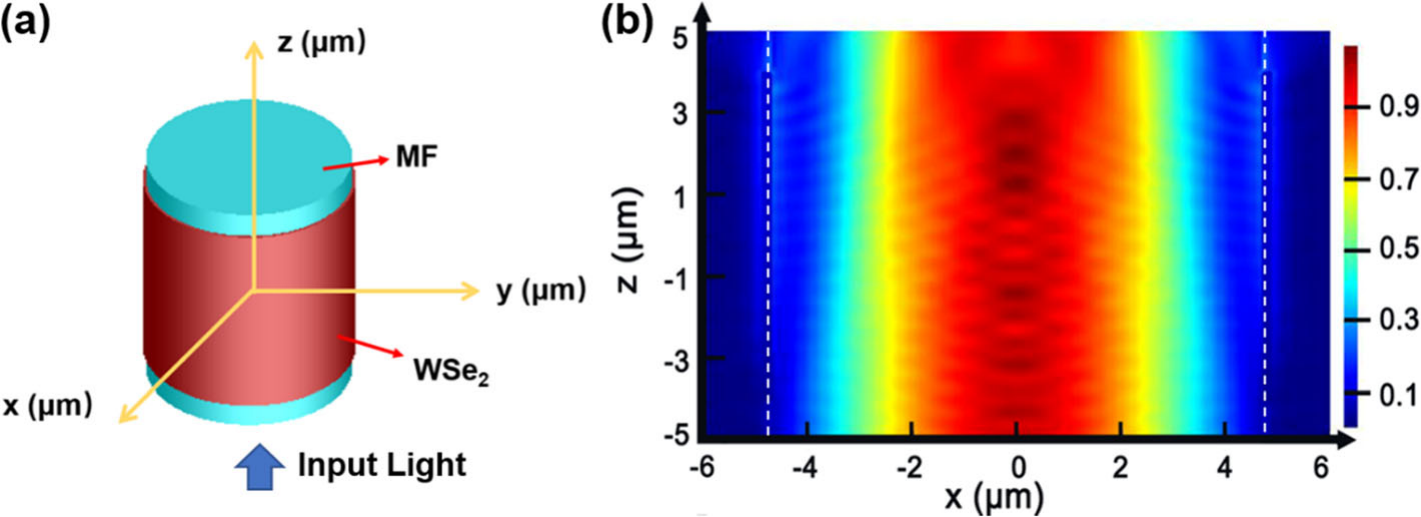
**a** The schematic of the device configuration for calculation with 3D FDTD. **b** The electric field distribution in the *x*-*z* plane cross section

**Fig. 17:**
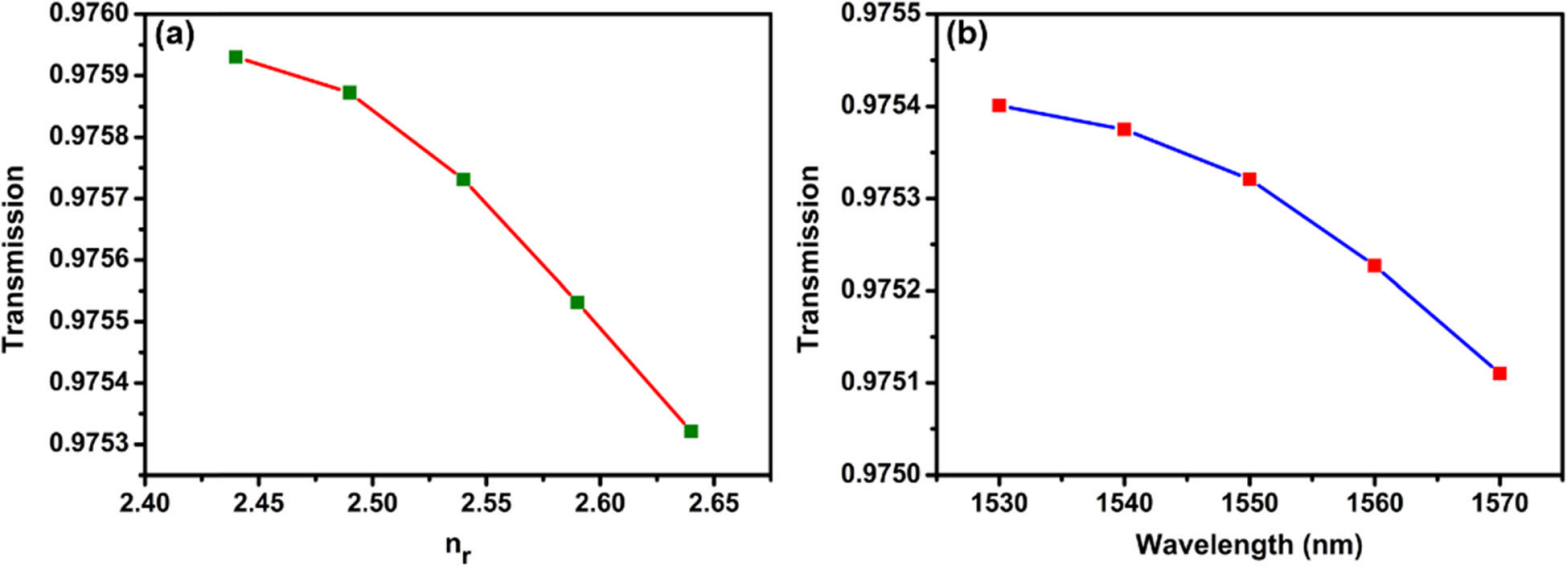
Calculated transmission as a function of **a** *n*_*r*_ and **b** wavelength

The 3D finite-difference time-domain (FDTD) (Lumerical FDTD Solution) was used to calculate the output power of the MF overlaid with WSe_2_. The schematic of the device configuration for output power calculation is shown in Fig. 16a. In the model, the thickness of WSe_2_ layer, the diameter of MF, and the refractive index of the MF were set to be 150 nm, 9.5 μm, and 1.46, respectively. The length of the MF is set to be 10 μm for qualitative calculation. The *x*, *y* and *z* directions have a grid resolution of 10 nm. The electric field distribution in the *x*-*z* plane cross-sectional cut at *y* = 0 μm is shown in Fig. 16b. The calculated transmission is shown in Fig. 17. As shown in Fig.17a, the transmission of the MF decreases with *n*_*r*_, and the variation trend is consistent with the results obtained with COMSOL. The losses are 10.80 and 10.94 dB/mm for *n*_*r*_ = 2.44 and *n*_*r*_ = 2.64, respectively. Then the transmission of MF for wavelengths from 1530 to 1570 nm was calculated with refractive index of WSe_2_ nanosheet fixed at 2.64 + 0.2i. As shown in Fig.17b, the transmission decreases with wavelength. The loss varied from 10.58 to 10.85 dB/mm when the wavelength changed from 1530 to 1570 nm.

The performance of light–control-light devices are compared in terms of TOP sensitivity and response time at different pump light wavelengths, as listed in Table 1. The all-optical control of light structure demonstrated here has higher sensitivity compared with the MF, MKR, and side-polished fiber (SPF) combined with various materials. The MF coated with WSe_2_ has faster response than the all-optical tuning structures such as MKR combined with WS_2_, MF overlaid with MoSe_2_, SPF combined with liquid crystals, and MF covered with WS_2_. Different factors contribute to higher TOP sensitivity and faster response time of MF overlaid with WSe_2_. Firstly, the WSe_2_ provides broad absorption bandwidth in the visible light and thermo-optic effect for all-optical tuning. Secondly, the MF structure is optimized for enhancing the light-matter interaction. Thirdly, the WSe_2_ nanosheets coating method enables precise nanosheet thickness control and uniform material deposition.

**Table 1:** Comparison of different light**-**control**-**light devices

Type of structure	Wavelength of pump light (nm)	Sensitivity (dB/mW)	Response time (s)	Reference
MF + bi–layer grapheme	1060	0.007	1 × 10^–6^	[8]
MF + graphene	980	0.20	NA	[9]
MKR + graphene	1530	0.02	NA	[13]
MKR + WS_2_	405	0.40	0.10	[14]
MF + reduced graphene oxide	405	0.21	NA	[16]
MF + MoSe_2_	405	0.165	0.60	[17]
MF + TiO_2_	405	0.28	NA	[31]
SPF + liquid crystals	458	0.15 at 25 °C	5.00	[44]
MF + WS_2_	660	0.136	0.42	[45]
MF + WSe_2_	405	0.30	15.3 × 10^–3^	This paper

## Conclusions

We have fabricated and demonstrated all-optical tuning of light in WSe_2_-coated MF based on the interaction between external pump light and WSe_2_. Through the external irradiation of pump light (405, 532, and 660 nm), WSe_2_’s broad absorption bandwidth and thermo-optic effect promise effective index change and subsequently output power variation. The sensitivity and fall time of 0.30 dB/mW and 15.3 ms can be obtained, respectively. The tuning mechanism of TOP is investigated with simulations. The performance of the MF covered with WSe_2_ such as TOP sensitivity and response time can be further improved by using monolayer thin film, modern nanofabrication methods, and optimized MF dimensions. The work is expected to promote WSe_2_’s realistic applications in all-optical modulator, multi-dimensionally tunable optical devices, etc.

## Data Availability

All data are fully available without restriction.

## Abbreviations

2D: Two-dimensional
DFB: Distributed feedback laser; SEM Scanning electron microscopy
MF: Microfiber
MKR: Microfiber knot resonator
*n*_eff_: Effective refractive index
*n*_*r*_: Refractive index
SPF: Side-polished fiber
TMDs: Transition metal dichalcogenides
TOP: Transmitted optical power

## References

[1] Amiri IS, Azzuhri S, Jalil MA, Hairi HM, Ali J, Bunruangses M, Yupapin P (2018). Introduction to photonics: principles and the most recent applications of microstructures. Micromachines.

[2] Li H, Yin ZY, He QY, Huang X, Lu G, DWH F, AIY T, Zhang Q, Zhang H (2012). Fabrication of single– and multilayer MoS_2_ film–based field–effect transistors for sensing no at room temperature. small.

[3] Wang QH, Kalantar–Zadeh K, Kis A, Coleman JN, Strano MS (2012). Electronics and optoelectronics of two–dimensional transition metal dichalcogenides. Nat. Nanotechnol.

[4] Radisavljevic B, Radenovic A, Brivio J, Giacometti V, Kis A (2011). Single–layer MoS_2_ transistors. Nat. Nanotechnol.

[5] Wang GZ, Liang GX, BAKER-MURRAY AIDANA, Wang KP, Wang JJ, Zhang XY, Bennett D, Luo JT, Wang J, Fan P, Blaud WJ (2018). Nonlinear optical performance of few-layer molybdenum diselenide as a slow-saturable absorber. Photonics Res..

[6] Bertolazzi S, Krasnozhon D, Kis A (2013). Nonvolatile Memory cells based on MoS_2_ graphene heterostructures. ACS Nano.

[7] Yu S, Meng C, Chen B, Wang H, Wu X, Liu W, Zhang S, Liu Y, Su Y, Tong L (2015). Graphene decorated microfiber for ultrafast optical modulation. Opt. Express.

[8] Liu ZB, Feng M, Jiang WS, Xin W, Wang P, Sheng QW, Liu YG, Wang DN, Zhou WY, Tian JG (2013). Broadband all-optical modulation using a graphene–covered–microfiber. Laser Phys. Lett..

[9] Chen JH, Zheng BC, Shao GH, Ge SJ, Xu F, Lu YQ (2015). An all–optical modulator based on a stereo graphene–microfiber structure. Light: Sci. Appl..

[10] Chen Z, Hsiao Vincent KS, Li X, Li Z, Yu J, Zhang J (2011). Optically tunable microfiber–knot resonator. Opt. Express.

[11] Zhou S, Wang Y, He D, Hu Y, Yu J, Chen Z, Guan H, Zhang J, Luo Y, Tang J, Lu H (2016). Add–drop filter based on wavelength–dependent light interlink between lithium–niobate microwaveguide chip and microfiber knot ring. Crystals.

[12] Wang P, Gu F, Zhang L, Tong L (2011). Polymer microfiber rings for high–sensitivity optical humidity sensing. Appl. Opt..

[13] Meng YH, Deng L, Liu ZL, Xiao HF, Guo XN, Liao MM, Guo AQ, Ying TH, Tian YH (2017). All–optical tunable microfiber knot resonator with graphene–assisted sandwich structure. Opt. Express.

[14] Chen GW, Zhang ZJ, Wang XL, Li HG, Jiang MJ, Guan HY, Qiu WT, Lu HH, Dong JL, Zhu WG, Yu JH, Zhong YC, Luo YH, Zhang J, Chen Z (2018). Highly sensitive all–optical control of light in WS_2_ coated microfiber knot resonator. Opt. Express.

[15] Lu HH, Wang ZM, Huang ZJ, Tao J, Xiong HQ, Qiu WT, Guan HY, Dong HZ, Dong JL, Zhu WG, Yu JH, Zhong YC, Luo YH, Zhang J, Chen Z (2018). Resonance–assisted light–control–light characteristics of SnS_2_ on a microfiber knot resonator with fast response. Photonics Res..

[16] Li DQ, Chen Z, Chen GL, Hu S, Wang YT, Qiu WT, Dong JL, Yu JH, Zhang J, Tang JY, Luo YH, Guan HY, Lu HH (2017). Reduced graphene oxide wrapped on microfiber and its light–control–light characteristics. Opt. Express.

[17] Zhang D, Guan H, Zhu W, Yu J, Lu H, Qiu W, Dong J, Zhang J, Luo Y, Chen Z (2017). All light–control–light properties of molybdenum diselenide (MoSe_2_)–coated–microfiber. Opt. Express.

[18] Revolinsky E, Beerntsen D (1964). Electrical properties of the MoTe_2_–WTe_2_ and MoSe_2_–WSe_2_ systems. J. Appl. Phys.

[19] Chiritescu C, Cahill DG, Nguyen N, Johnson D, Bodapati A, Keblinski P, Zschack P (2007). Ultralow thermal conductivity in disordered, layered WSe_2_ crystal. Science.

[20] Baugher BW, Churchill HO, Yang Y, Jarillo–Herrero P (2014). Optoelectronic devices based on electrically tunable p–n diodes in a monolayer dichalcogenid. Nat. Nanotechnol.

[21] Seyler KL, Schaibley JR, Gong P, Rivera P, Jones AM, Wu SF, Yan JQ, Mandrus DG, Yao W, Xu XD (2015). Electrical control of second–harmonic generation in a WSe_2_ monolayer transistor. Nat. Nanotechnol.

[22] Lewerenz HJ, Heller A, Disalvo FJ, Chem JA (1980). Relationship between surface morphology and solar conversion efficiency of \VSe_2_ photoanodes. Am. Chem. J.

[23] Liu W, Kang J, Sarkar D, Khatami Y, Jena D, Banerjee K (2013). Role of metal contacts in designing high performance monolayer n–type WSe_2_ field effect transistors. Nano Lett..

[24] Podzorov V, Gershenson ME, Kloc C, Zeis CR, Bucher E (2004). High–mobility field–effect transistors based on transition metal dichalcogenides. Appl. Phys. Lett..

[25] Chuang HJ, Tan XB, Ghimire NJ, Perera MM, Chamlagain B, Cheng MC, Yan JQ, Mandrus D, Tománek D, Zhou ZX (2014). High mobility WSe2 p– and n–type field–effect transistors contacted by highly doped graphene for low–resistance contacts. Nano Lett..

[26] Tonndorf P, Schmidt R, Böttger P, Zhang X, Börner J, Liebig A, Albrecht M, Kloc C, Gordan O, Zahn DRT, de Vasconcellos M, Bratschitsch R (2013). Photoluminescence emission and Raman response of monolayer MoS_2_, MoSe_2_, and WSe_2_. Opt. Express.

[27] Chen BH, Zhang XY, Wu K, Wang H, Wang J, Chen JP (2015). Q–switched fiber laser based on transition metal dichalcogenides MoS_2_, MoSe_2_, WS_2_, and WSe_2_. Opt. Express.

[28] Chen GY, Ding M, Newson TP, Brambilla G (2013). A review of microfiber and nanofiber based optical sensors. The Open Optics Journal.

[29] Tong LM, Zi F, Guo X, Lou JY (2012). Optical microfibers and nanofibers: a tutorial. Opt. Commun.

[30] Allsop T, Floreani F, Jedrzejewski KP (2016). Spectral characteristics of tapered LPG device as a sensing element for refractive index and temperature. J. Lightwave Technol..

[31] He D, Hu Y, Tao J, Zheng X, Liu H, Jing G, Lu H, Guan H, Yu J, Zhang J, Tang J, Luo Y, Chen Z (2017). Micro fiber with cladding of titanium dioxide (TiO_2_) nanoparticles and its violet light sensing. Opt. Mater. Express.

[32] Wo JH, Wang GH, Cui Y (2012). Refractive index sensor using microfiber–based Mach–Zehnder interferometer. Opt Lett..

[33] Wu Q, Chen S, Wang YZ, Wu LM, Jiang XT, Zhang F, Jin XX, Jiang QY, Zheng Z, Li JQ, Zhang M, Zhang H (2019). MZI-based all-optical modulator using MXene Ti_3_C_2_T_x_ (T = F, O, or OH) deposited microfiber. Adv. Mater..

[34] Song Yufeng, Liang Zhiming, Jiang Xiantao, Chen Yunxiang, Li Zhongjun, Lu Lu, Ge Yanqi, Wang Ke, Zheng Jilin, Lu Shunbin, Ji Jianhua, Zhang Han (2017). Few-layer antimonene decorated microfiber: ultra-short pulse generation and all-optical thresholding with enhanced long term stability. 2D Materials.

[35] Liu M, Zheng XW, Qi YL, Liu H, Luo AP, Luo ZC, Xu WC, Zhao CJ, Zhang H (2014). Microfiber-based few-layer MoS_2_ saturable absorber for 2.5 GHz passively harmonic modelocked fiber laser. Opt. Express.

[36] Sahin H, Tongay S, Horzum S, Fan W, Zhou J, Li J, Wu J, Peeters MF (2013). Anomalous Raman spectra and thickness–dependent electronic properties of WSe_2_. Phys. Rev. B.

[37] Kleemann ME, Chikkaraddy R, Alexeev EM (2017). Strong-coupling of WSe_2_ in ultra-compact plasmonic nanocavities at room temperature. Nature Communications.

[38] Yu S, Wu X, Wang Y, Guo X, Tong L (2017). 2D Materials for optical modulation: challenges and opportunities. Advanced Materials.

[39] Sun QZ, Sun XH, Jia WH, Xu Z, Luo HP, Liu DM, Lin Z (2016). Graphene-assisted microfiber for optical-power-based temperature sensor. IEEE Photonics Technology Letters.

[40] Xu K, Xu C, Xie YY, Deng J, Zhu YX, Guo WL, Xun M, Teo KBK, Chen HD, Sun J (2015). Graphene GaN-Based Schottky Ultraviolet Detectors. IEEE Trans. Electron Devices.

[41] Sun Q, Sun X, Jia W, Xu Z, Luo H, Liu D, Zhang L (2016). Graphene–assisted microfiber for Optical–Power–Based temperature sensor. IEEE Photonics Technol. Lett..

[42] Kleemann M, Chikkaraddy R, Alexeev EM, Kos D, Carnegie C, Deacon W (2017). Strong–coupling of WSe_2_ in ultra–compact plasmonic nanocavities at room temperature. Nat. Commun..

[43] Eichfeld SM, Eichfeld CM, Lin YC, Hossain L, Robinson JA (2014). Rapid, non-destructive evaluation of ultrathin WSe_2_ using spectroscopic ellipsometry. APL Materials.

[44] Hsiao VKS, Li Z, Chen Z, Peng P, Tang J (2009). Optically controllable side–polished fiber attenuator with photoresponsive liquid crystal overlay. Opt. Express.

[45] Li HG, Guan HY, Chen GW, Zhang ZJ, Wang XL, Jiang MJ, Lang YW, Zhu WG, Yu JH, Lu HH, Qiu WT, Dong JL, Luo YH, Zhang J, Chen Z (2018). Broadband light–control–light characteristics of WS_2_ on microfiber. International Society for Optics and Photonics.

